# Facile Synthesis of Gallium (III)-Chitosan Complexes as Antibacterial Biomaterial

**DOI:** 10.3390/pharmaceutics13101702

**Published:** 2021-10-15

**Authors:** Muhammad Asim Akhtar, Zoya Hadzhieva, Kanwal Ilyas, Muhammad Saad Ali, Wolfgang Peukert, Aldo R. Boccaccini

**Affiliations:** 1 Department of Materials Science and Engineering, Institute of Biomaterials, University of Erlangen-Nuremberg, 91058 Erlangen, Germany; asim.akhtar@fau.de (M.A.A.); zoya.hadzhieva@fau.de (Z.H.); kanwal.ilyas@fau.de (K.I.); 2 Department of Chemical and Biological Engineering, Institute of Particle Technology, University of Erlangen-Nuremberg, 91058 Erlangen, Germany; muhammad.saad.ali@fau.de (M.S.A.); Wolfgang.peukert@fau.de (W.P.)

**Keywords:** chitosan, complexes, chelate, antibacterial, gallium, biomaterial

## Abstract

Even though antibiotic treatment remains one of the most common tools to handle bacterial infections, the excessive antibiotic concentration at the target site may lead to undesired effects. Aiming at the fabrication of antibiotic-free biomaterials for antibacterial applications, in this work, we propose the synthesis of gallium (III)—chitosan (Ga (III)-CS) complexes with six different gallium concentrations via an in situ precipitation method. Fourier Transform infrared spectroscopy indicated the chelation of chitosan with Ga (III) by peak shifts and changes in the relative absorbance of key spectral bands, while energy-dispersive X-ray spectroscopy indicated the homogenous distribution of the metal ions within the polymer matrix. Additionally, similar to CS, all Ga (III)-CS complexes showed hydrophobic behavior during static contact-angle measurements. The antibacterial property of the complexes against both Gram-negative and Gram-positive bacteria was positively correlated with the Ga (III) concentration. Moreover, cell studies confirmed the nontoxic behavior of the complexes against the human osteosarcoma cell line (MG-63 cells) and mouse embryonic fibroblasts cell line (MEFs). Based on the results of this study, new antibiotic-free antibacterial biomaterials based on Ga (III)-CS can be developed, expanding the scope of CS applications in the biomedical field.

## 1. Introduction

Infectious diseases represent devastating complications of implantation surgery, which could be associated with any biomaterial, regardless of type, form, function, or contact duration with human tissue [1,2]. Even though the systemic delivery of antibiotics is a conventional approach to combat fatal infections, this practice brings potential disadvantages, including low drug concentration at the target site, need for hospitalized monitoring, and antibiotic resistance of microorganisms [1,2,3]. Thus, in order to prevent the formation of biofilms and to avoid the use of antibiotics, new antimicrobial material strategies need to be developed [4].

Chitosan (CS) is a biopolymer that demonstrates antimicrobial activity against a wide range of microorganisms [5]. In addition, this polymer is characterized by numerous other beneficial biological properties, such as antitumor and antioxidant activities, biocompatibility, and biodegradability [6]. One of the key properties of CS is its ability to create complexes with wide range of metal ions [7]. The main applications of the metal binding property of CS or its derivatives are in wastewater and dye purification [8,9,10,11]. There is also increasing interest to use the advantageous properties of chitosan-metal ion complexes in the biomedical field. Gritsch et al. [12], for instance, implemented a chitosan-copper metal ion complex to develop antibacterial (and angiogenic) polymers, exploiting the effects of Cu as a bioinorganic element, without using potentially problematic as well as expensive growth factors or sensitive molecules. Additionally, the chelation ability of CS has been recently exploited against metal-induced dermatitis, since chitosan-based gels were able to reduce the penetration of allergic metal ions (Ni, Cr, Co, or Pd) through the epidermis [13]. The entrapment of specific antimicrobial ions (Cu (II), Zn (II), Fe (II), Zr (IV), or Ag (I)) in the CS matrix is also frequently applied to improve the antibacterial properties of materials, mainly for high-tech wrapping applications [14,15,16]. However, metals, such as Cu, Zn, or Ag, can be problematic due to their ability to accumulate in the food chain or vital organs in the human body [17]. In addition, there is still limited research focused on the binding property of unmodified CS with trivalent antibacterial metallic ions to design antibiotic-free biomaterials with antimicrobial potential. Therefore, in this work, we utilized the chelation ability of CS with metal ions, in particular focusing on the therapeutic trivalent metal ion gallium (Ga). Gallium can interact with cells and proteins due to its resemblance with iron ion. However, there is no proven physiological function of Ga in biological systems [18,19]. Ga (III) is reported to exert inhibitory efficiency against numerous bacteria (*S. aureus*, *P. aeruginosa*, *E. coli*, *A. baumannii*, etc.), as well as therapeutic activity against diverse disorders, including accelerated bone resorption, autoimmune disease, and certain cancers [20,21,22,23]. According to Pearson’s classification from 1967, Ga (III) ion bonds most strongly to oxygen atoms and, to a lower magnitude, with nitrogen atoms on ligands [20]. This behavior makes Ga a suitable candidate for complexation with CS through bonding with N and O atoms from the amino and hydroxyl groups on the polymer chain and at least one water molecule, probably forming penta- or hexacoordinative complexes similar to Fe^3+^ [11,24,25]. Previous works have emphasized the application of gallium-chitosan complexes for intertumoral radiotherapy [26] or orthopedic implant coatings [27]. Nevertheless, no systematic studies on the synthesis approach, as well as the structural, physical, and biological characterization of the raw complex material have been reported.

In this work, we present for the first time a versatile and reproducible way to synthesize Ga (III)-CS complexes with intrinsic antibacterial properties and suitable biocompatibility. We anticipate that the promising results of this study will encourage in future the implementation of Ga (III)-CS complex in biomedical devices, such as functional coatings for implants [27], wound dressing, or as tissue engineering scaffolds.

## 2. Experimental

### 2.1. Materials

CS (molecular weight: 190–310 kDa, degree of deacetylation: 75–85%), gallium (III) nitrate hydrate, and acetic acid (99 %) from Sigma Aldrich (Taufkirchen, Germany) were used, in accordance with previously published studies on the fabrication of chitosan-metal ion complexes [12]. Ethanol (99 %) was obtained from VWR (Darmstadt, Germany).

### 2.2. Preparation Gallium (III)-Chitosan Complex

Ga (III)-CS complex films were synthesized via an in situ precipitation method, modifying previously described protocols [11,12]. First, 2 % (*w*/*v*) CS was dissolved in acetic acid solution with a concentration of 2 % (*v*/*v*) under constant stirring at 40 °C for 24 h. Different concentrations of gallium nitrate hydrate (Ga(NO_3_)_3_·nH_2_O) were added to the above-mentioned solution and left to disperse for 4 h. Samples with six different concentrations were produced (Table 1). The amount of gallium nitrate hydrate in the solution with respect to the theoretical amount of free amino groups of CS was calculated by using the following equations:

(1) $$m_{\mathrm{Ga}{\left({\mathrm{NO}}_{3}\right)}_{3}\cdot H_{2}O\;}=X\;\times {\;\mathrm{MM}}_{\mathrm{Ga}{\left({\mathrm{NO}}_{3}\right)}_{3}\cdot H_{2}O}\times \frac{m_{\mathrm{CS}}}{\bar{\mathrm{MM}}}$$

(2) $$\bar{\mathrm{MM}}=\mathrm{DDA}\times {\;\mathrm{MM}}_{\mathrm{glu}}+\left(1-\mathrm{DDA}\right)\times {\;\mathrm{MM}}_{N-\mathrm{acetylglu}}=187.578{\;g\;\mathrm{mol}}^{-1}$$

with:

$m_{\mathrm{Ga}{\left({\mathrm{NO}}_{3}\right)}_{3}.H_{2}O}:\;\mathrm{amount}\;\mathrm{of}\;\mathrm{gallium}\;\mathrm{nitrate}\;\mathrm{hydrate}\;\left[g\right];$
$X:\mathrm{fraction}\;\mathrm{of}\;\mathrm{Ga}\left(\mathrm{III}\right)\;\mathrm{ions}\;\mathrm{to}\;\mathrm{free}\;\mathrm{amino}\;\mathrm{groups}\;\left(\mathrm{Ga}\;\left(\mathrm{III}\right):{\mathrm{NH}}_{2}\right);\;$
${\mathrm{MM}}_{\mathrm{Ga}{\left({\mathrm{NO}}_{3}\right)}_{3}\cdot H_{2}O}:\;\mathrm{molecular}\;\mathrm{mass}\;\mathrm{of}\;\mathrm{gallium}\;\mathrm{nitrate}\;\mathrm{hydrate}\;=255.74{\;g\;\mathrm{mol}}^{-1};\;$
$m_{\mathrm{CS}}:\mathrm{weight}\;\mathrm{of}\;\mathrm{chitosan}\;\left[g\right];\;$
DDA:deacetylation degree of chitosan ∼80 % (the average of the given range); 
${\mathrm{MM}}_{\mathrm{glu}}:\;\mathrm{molecular}\;\mathrm{weights}\;\mathrm{of}\;\mathrm{glucosamine}\;\mathrm{moieties}=179.17{\;g\;\mathrm{mol}}^{-1};$
${\mathrm{MM}}_{N-\mathrm{acetylglu}}:\mathrm{molecular}\;\mathrm{weights}\;\mathrm{of}\;N-\mathrm{acetylglucosamine}\;\mathrm{moieties}\;=221.21{\;g\;\mathrm{mol}}^{-1}.$

**Table 1:** Composition of produced Ga (III)-CS complexes.

Ga (III): NH_2_	CS	1:32	1:16	1:8	1:4	1:2	1:1
X [%]	0	3	6	12	24	48	96
$m_{\mathrm{Ga}{\left({\mathrm{NO}}_{3}\right)}_{3}\cdot H_{2}O}\;\left[\mathrm{mg}\right]$	0	21	42	84	164	328	656

The complex solution was added in ethanol (ratio 1:2) to obtain the precipitates of Ga (III)-CS. After 2 h, the final products were repeatedly rinsed with ethanol to neutralize the pH and also to remove the excess of Ga(NO_3_)_3_·nH_2_O. The films were dried at 60 °C overnight. The compositions of the produced Ga (III)-CS complexes are listed in Table 1, where the labelling corresponds to the ratio of gallium ions to free amino groups (Ga(III): NH_2_). CS without gallium was used as the control.

### 2.3. Characterizations

#### 2.3.1. Morphological and Compositional Characterization

Scanning electron microscopy (FESEM, Auriga CrossBeam, Carl Zeiss Microscopy GmbH, Jena, Germany) was used to analyze the surface morphologies of the prepared complexes. Furthermore, energy dispersive X-ray (EDX) spectrometry and Fourier transform infrared spectroscopy (FTIR) (Shimadzu IRAffinity-1S spectrometer, Shimadzu Corp, Tokyo, Japan) were used to confirm the presence of gallium in the complexes. A 20 kV electron accelerating voltage was employed for EDX analysis, while the FTIR data were collected in absorbance mode from 400 to 4000 cm^−1^.

#### 2.3.2. X-ray Diffraction

The structural analysis of the complexes was done by using X-ray diffraction (XRD) (Miniflex 600, Rigaku Corporation, Neu-Isenburg, Germany) in the 2θ range of 10° to 80°. XRD patterns were obtained with the 2θ rate of 1° per minute with a step size of 0.010° at 40 kV by using Cu Kα radiation.

#### 2.3.3. Release Studies

Ion release characterization of the complexes was done by soaking the samples in PBS. Briefly, 10 mg of each type of Ga (III)-CS complexes were immersed in 10 mL of PBS at 37 °C. Then, 5 mL of PBS were removed at each given time point for elemental analysis and 5 mL of fresh PBS were refilled to keep a total volume of 10 mL. The ion concentration was measured using an inductively coupled plasma optical spectrometer (ICP-OES, Optima 8300, Perkin Elmer, Waltham, MA, USA). The device was calibrated using a 1000 mg/L Ga standard solution purchased from Carl Roth, Germany. The calibration was performed over a wide range of values using points at 0.5, 0.75, 1, 1.25, 1.5, 2, 5, 10, 25, 50, and 100 mg/L. The concentration was then calculated via weighted linear regression using a six-point calibration plot, considering the calibration points closest to the measured emission intensity.

#### 2.3.4. Wettability

Contact angle measurements were carried out with the DSA30 instrument (Kruess GmbH, Hamburg, Germany) by the sessile drop method to evaluate the wettability of Ga (III)-CS complex films. The contact angle was continuously measured for 3 min by dropping deionized water on the flat samples.

#### 2.3.5. Antibacterial Assay

The antibacterial activity of the complexes was evaluated against *Staphylococcus aureus* (*S. aureus*) and *Escherichia coli* (*E. coli*) through in vitro experiments. Prior to the measurements, samples were kept in UV light for 1 h for sterilization. In order to evaluate the sample’s antibacterial activity over a period of time, a colony-forming unit (CFU) counting was done. Lysogeny broth (LB) medium was used to cultivate both bacteria overnight at 37 °C. A bacterial suspension with an optical density of 0.015 that contains ∼1 × 10^7^ CFU/mL was prepared. This diluted bacterial suspension was added to each sample in each well of a 24-well plate. The sample weight to bacterial suspension was kept constant, i.e., 10 mg/mL. The plate was incubated under static conditions at 37 °C overnight. The growth medium was withdrawn and diluted 10^6^ times in order to count the bacterial colonies on agar plates. To observe bacterial inhibition and/or growth, 30 µL of the diluted medium were spread onto a Luria-Bertani (LB) agar plate and incubated overnight. The following day, the agar plates were evaluated by optical images and bacterial colonies were counted by using ImageJ 1.5i software (National Institutes of Health, Bethesda, MD, USA). CFU/mL was calculated by using the following formula:

(3) $$\frac{\mathrm{CFU}}{\mathrm{ml}}=\frac{\mathrm{no}.\;\mathrm{of}\;\mathrm{colonies}\;\times \;\mathrm{dilution}\;\mathrm{factor}}{\mathrm{volume}\;\mathrm{of}\;\mathrm{culture}\;\mathrm{plate}}$$

#### 2.3.6. Cell Studies

Cell culture studies were done using the human osteoblast-like cell line (MG-63) and mouse embryonic fibroblasts cell line (MEFs) (Sigma-Aldrich, Taufkirchen, Germany) by an indirect method. Initially, the complexes and control (CS) were sterilized under UV irradiation for 1 h. All investigated samples were immersed in Dulbecco’s modified Eagle’s medium (DMEM, Gibco, Schwerte Germany) with the same concentration used in the case of the antibacterial studies, i.e., 10 mg/mL for 24 h at 37 °C. The DMEM contained 10 vol. % fetal bovine serum and 1 vol. % penicillin/streptomycin (Sigma-Aldrich, Taufkirchen, Germany). First, 5 × 10^4^ cells were seeded in each well and placed in a static incubator for 24 h. After, cultured medium was removed and replaced with extract from the pre-incubated complexes and control samples. Cells were incubated again for another 24 h. The cell viability was calculated by using water-soluble tetrazolium salt (WST-8 assay kit, Sigma-Aldrich, Taufkirchen, Germany). After 24 h of incubation, 1 vol% solution of WST-8 in DMEM was added in the samples and further incubated for 3 h at 37 °C. Finally, the absorbance at 450 nm was measured and cell viability (%) was calculated by using the following equation:

(4) $$\mathrm{Cell}\;\mathrm{viability}\;\left[\%\right]=\frac{{\mathrm{OD}}_{\mathrm{sample}}-{\mathrm{OD}}_{\mathrm{blank}}}{{\mathrm{OD}\;}_{\mathrm{reference}}-{\mathrm{OD}}_{\mathrm{blank}}}\times 100$$

where OD_sample_ is the absorbance of each specimen, OD_blank_ is the absorbance of the WST reactant, and OD_reference_ is the absorbance of the respective positive control.

To investigate their morphology, cells were stained with Calcein AM (calcein acetoxymethyl ester) and DAPI (4′,6-diamidino-2-phenylindole, dilactate) (Life Technologies, Darmstadt, Germany) and imaged by using a fluorescence microscope (Axio Scope A1, Carl Zeiss Microimaging GmbH, Jena, Germany).

### 2.4. Statistical Analysis

The assessment between the results was done by using the one-way ANOVA test with Bonferroni’s post-hoc test and *p* values < 0.05 were considered statistically significant. Significant differences are represented by an asterisk (*). The results are presented by the mean value ± standard deviation (SD).

## 3. Results and Discussion

### 3.1. Morphological and Compositional Characterization

**Figure 1:**
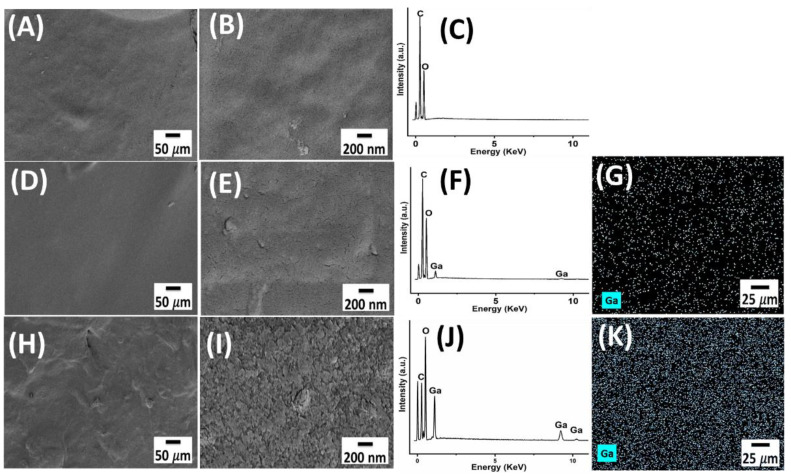
(**A**,**B**) SEM micrographs of CS at two magnifications, (**C**) EDX spectra of the respective film, (**D**,**E**) SEM micrographs of 1:8 Ga (III)-CS complex film at two magnifications, (**F**,**G**) EDX spectra and mapping of Ga in the 1:8 Ga (III)-CS complex film, (**H**,**I**) SEM micrographs of 1:1 Ga (III)-CS complex film at two magnifications, and (**J**,**K**) EDX spectra and mapping of Ga in 1:1 Ga (III)-CS complex film.

The SEM images of CS and Ga (III)-CS complex films at two different magnifications are shown in Figure 1**.** For reasons of clarity, only results for CS, 1:8, and 1:1 Ga (III)-CS complex are presented. At low magnifications, the surface of all Ga (III)-CS complex films appears smooth and homogenous, similar to the pure CS samples. As suggested by Trimukhe et al. [28], this is an indication of the strong and specific binding of amino groups of CS with the metal ions. Within the detection limit of XRD analysis and under SEM magnification, no crystalline structures were detected, which indicates that coordination bonds formed between the polymer and Ga (III). However, even though the addition of gallium ions did not cause any qualitative changes in the microstructure of the samples, high magnification images showed an increase in roughness with a higher Ga amount (Figure 1B,E,I). This high roughness was observed in 1:4, 1:2, and 1:1 of Ga (III)-CS complexes. However, at lower concentrations (1:32, 1:16, and 1:8), the surfaces of these films were smooth and homogeneous. The reason for this phenomenon is likely the fact that an excess concentration of Ga (III) ions can cause increased chelation with the polymer matrix. As a result, metal uptake occurs near the surface since the metal ions do not penetrate into the inner part of the polymer network [29], which can lead to the observed nanoscaled roughness. However, the formation of (amorphous) nanoprecipitates (e.g., Ga-hydroxides) cannot be ruled out as these would not be detectable by the applied techniques (SEM, XRD) and should be investigated in the future.

The EDX spectra of all investigated samples (Figure 1C,F,J) showed carbon and oxygen peaks, which are related to the main constituting elements of CS [12]. The EDX spectra of chitosan-metal complexes additionally demonstrated the presence of gallium ions, as with increasing metal ion quantity, the relative intensity of the K and L peaks also increased. Gallium mappings across the surface of the samples revealed that gallium was homogeneously distributed in the CS matrix for all studied formulations (Figure 1G,K). No significant contamination from laboratory tools or residual initial gallium salt was detected.

The amount of gallium in the complexes was semi-quantitatively assessed by the area ratios of the gallium L (Ga_Lα_) and the carbon (C_k__α_) peaks from several spectra (n = 4) (Table 2). It is visible that the resulting Ga_Lα_/C_kα_ ratio increased with the higher concentration of Ga (III) in the complex, confirming increased chelation of gallium ions [12].

**Figure 2:**
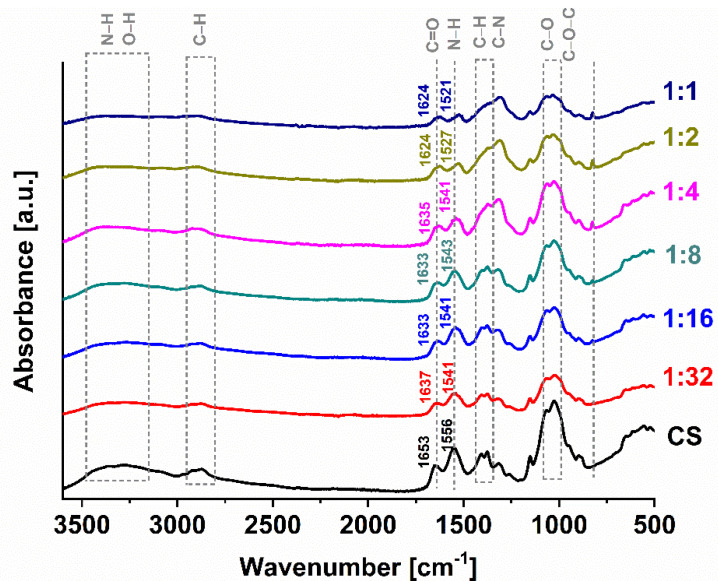
FTIR spectra of CS and Ga (III)-CS complexes. The related bands are discussed in the text.

The structure of CS is modified due to the complexation with Ga(III), which is expressed by the change in the relative absorbance and wavenumbers of particular bands. The FTIR spectra of the CS and all Ga (III)-CS complexes are shown in Figure 2. In the range of 3600–3000 cm^−1^, CS exhibited a broad band, which is attributed to -NH_2_ stretching vibration and asymmetrical stretching vibration of –OH [11,30]. The characteristic peak of C-H asymmetric stretching vibration can be seen at 2875 cm^−1^. The peaks at 1650 and 1550 cm^−1^ represent the stretching of the C=O group of amide I band and -NH bending vibrations of amide II band, respectively [12,30,31]. The deformation of C-H and the stretching of C-N generally appears around 1400 cm^−1^ [12]. The presence of C-O and C-O-C can be confirmed by the 1150–1000 cm^−1^ absorption bands. The occurrence of the absorption peak at 895 cm^−1^ suggests that the CH of the β-glycosidic bond is deformed [30,32].

In case of all investigated Ga (III)-CS complexes, a reduction in the relative absorbance at 3300 cm^−1^ was observed, which was caused by the reduction in the electron density of -NH_2_ and -OH bonds and decrease in the bond force constant of CS bonding due to the complexation [33]. In addition, the complexes exhibited amide I, II, and III band shift to lower frequencies, and the shift generally increased with the higher Ga (III) amount. The relative peaks intensities of amide and amine at 1650 cm^−1^ and 1550 cm^−1^ could also be detected. All mentioned, modifications of the spectral bands confirm that both hydroxyl and amine groups play a role in chelation [12,30,34]. The presence of Ga (III) in the CS matrix changed the intensity and shape of C-H stretching and bending peaks at 2880 and 1410 cm^−1^. The changes in C-H stretching and bending peaks at 2880 and 1410 cm^−1^ in terms of peak shape and intensity caused by the change in the environment of CH_2_OH due to Ga (III) in the CS structure [35,36]. Furthermore, the line shape of the glycosidic bond characteristic peak at ∼ 1100 cm^−1^ changed, evidencing the stretching of the glucosamine monomers of CS chains due to the incorporation of Ga (III) [12]. The new peak, appearing at 825 cm^−1^ after metal ion addition, can be ascribed to the stretching vibration of the Ga (III)-CS complexes and shows increasing absorbance with a higher gallium concentration [31].

In the literature, two models for the coordination bond between CS and metal ions are discussed, namely the bridge model and the pendant model [10]. According to the obtained FTIR spectra, it can be speculated that in all Ga (III)-CS complexes, Ga (III) is coordinated between different CS chains due to the variation in the peak of the glycosidic bond (1100 cm^−1^). This band modification is typical for the bridge model, in which gallium ion is bound to two amino groups from two separated polymer chains [12].

According to the literature, Fe (III) forms hexacoordinated bonds with CS molecules and complexation is primarily due to the amino and hydroxyl groups of CS. Moreover water molecules in solution would participate in the coordination sphere of CS-Fe(III) complex [11,37]. Due to the chemical and physical similarity of Ga (III) and Fe (III), we expect that Ga (III) forms complexes with CS in the same way as Fe (III). Nevertheless, there is a variety of mechanisms of chelation, so that additional structural analysis is required to fully characterize the interaction of Ga ions with CS [38]. The proposed chemical structures of all investigated complexes are given in the Supplementary Material. A complete survey of this topic is beyond the scope of this study.

### 3.2. Confirmation of Complexation by XRD

**Figure 3:**
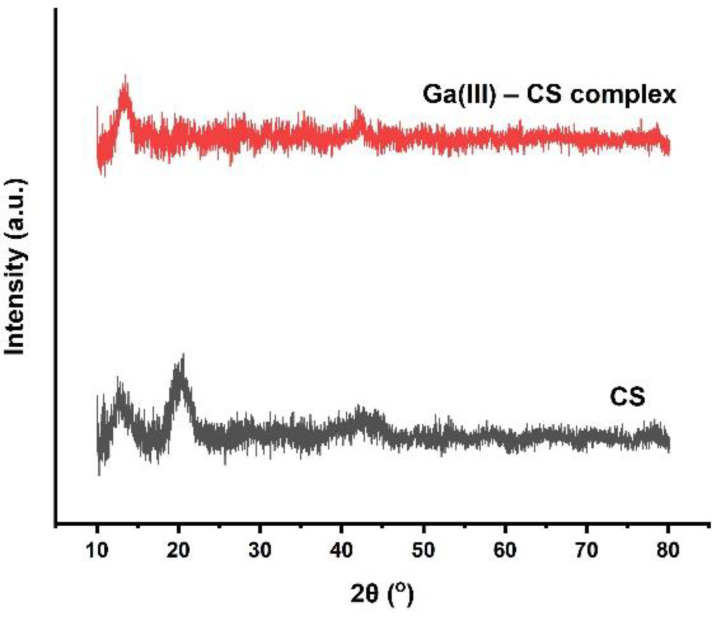
Comparison between XRD patterns of CS and 1:1 Ga (III)-CS complex. All other formulations behaved similarly. Changes in the XRD pattern with addition of Ga (III) are discussed in the text.

As discussed, complexation brings clear changes in the characteristic peaks of CS in FTIR spectra. In addition, XRD analyses were performed, and the patterns are shown in Figure 3. It can be observed that the semi-crystalline nature of CS is confirmed by two broad characteristic fingerprint diffraction peaks at 2θ = 12° and 2θ = 20° [25,39]. After the addition of gallium, the peak at 2θ = 20° disappeared, and the same was observed for all Ga (III)-CS complexes, without significant differences among complexes with different concentrations of Ga (III). Chelation of CS with metal ions change the crystallinity of CS [39]. Complexation of CS with metal ions decreases the available binding sites for hydrogen bonding in amine and hydroxyl groups. This phenomenon brings a decrease in the number of inter- and intramolecular bonds between CS chains, which take part in the self-assembly of the polysaccharide [25]. A small peak at 2θ = 42° can be observed after complexation. Information in the literature regarding this observed XRD peak is very limited. However, Gritsch et al. [39] suggested that this peak might be due to the new crystal conformation of CS formed as a consequence of the complexation. Moreover, no peak related to gallium was observed in the XRD patterns. From these results, it can be proposed that Ga (III) formed a complex without precipitating out as a second phase. This change in the characteristic peaks of CS has also been reported in the literature for copper (II)-CS as well as Fe (III)-CS complexes [25,39,40].

### 3.3. Ion Release from Complexes

**Figure 4:**
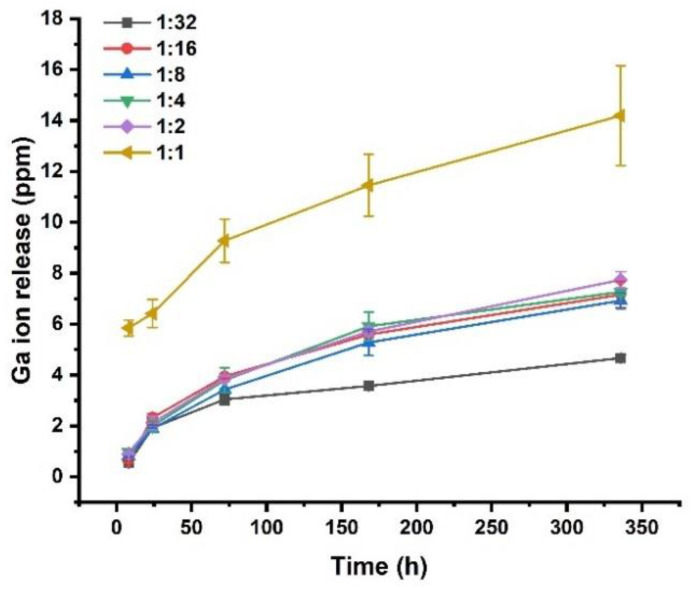
Cumulative ion release profile of gallium from different Ga (III)-CS complexes in PBS.

The release of gallium from different Ga (III)-CS complexes was quantified using ion release tests and the results are shown in Figure 4. The 1:1 Ga (III)–CS complex showed the highest amount of gallium release compared to other complexes at each time point. As mentioned earlier, the high amount of metal ions did not infiltrate into the inner part of the CS network and metal uptake occurred near the surface. As a result, unbound surface metal ions are released at a higher rate. Interestingly, a concentration-dependent release profile was not observed. Apart from the 1:1 Ga (III)–CS sample, all complexes showed the same amount of gallium release up to 24 h. It is important to note that the release study was done in saline solution (PBS). The initial release of gallium would be due to the swelling mechanism of CS. We expect that a significant amount of gallium is still available in the CS films and will be released once the polymer is resorbed. CS degrades enzymatically by hydrolyzing glucosamine–glucosamine and N-acetyl-glucosamine–N-acetylglucosamine linkages [41]. It is well documented that CS can absorb metal ions from water and form hexa-coordinative and tetra-coordinative complexes in the case of trivalent and divalent ions, respectively [11,42,43,44]. Based on this property, we can expect that in saline solution, CS does not allow a concentration-dependent gallium ion release. In future studies, an ion release study including enzymatic degradation should be done. In that case, it would be possible to compute the long-term release of gallium from Ga (III)–CS complexes. Still, the prepared films exhibited a sustained release of the therapeutic ions with time, which is favorable for preventing potential bacterial colonization of the biomaterial following implantation.

### 3.4. Wettability

**Figure 5:**
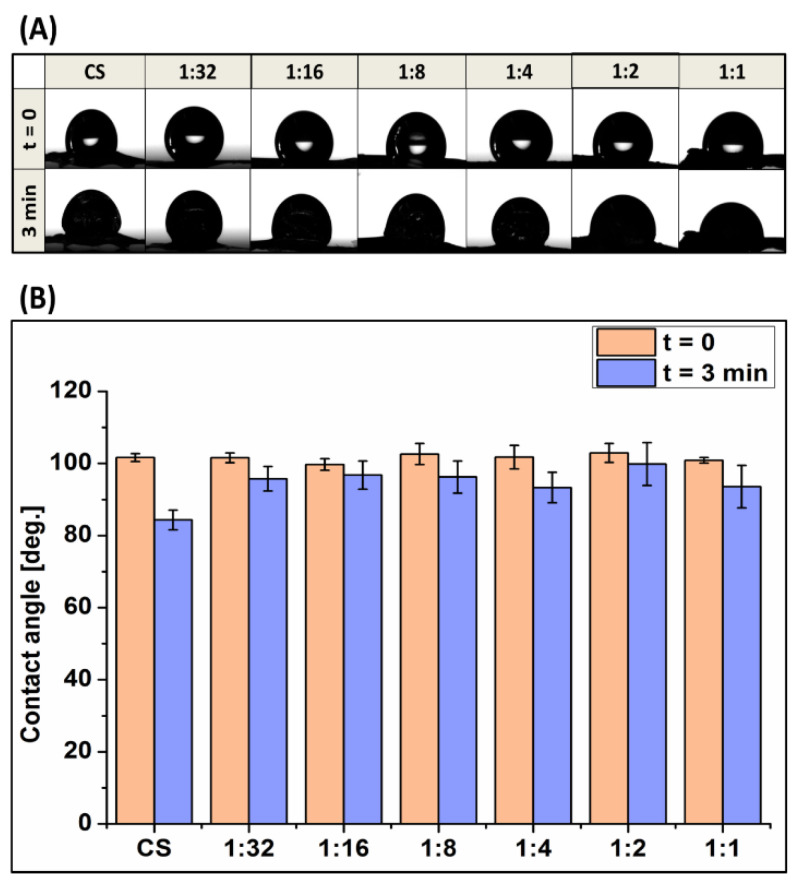
(**A**) Profiles of water droplets on CS and Ga (III)-CS samples, (**B**) average contact angle of water droplets on all investigated samples, immediately and 3 min after deposition.

The contact angle values of Ga (III)-CS immediately after droplet deposition on the surface and after 3 min are presented in Figure 5B. The measurements showed that the addition of Ga caused no statistically significant differences (*p* > 0.05) in initial wettability, regardless of the amount of Ga. Pure CS had an initial contact angle of 104 ± 4°, which is close to values published in the literature [45]. Similar outcomes were measured for all Ga (III)-CS immediately after deposition. After 3 min, droplets on pure CS quickly reduced their angle to 84 ± 2° due to the spreading of the liquid on the surface. The same decreasing trend with time was observed for samples modified with gallium ions, but the reduction of the starting contact angle was attenuated. This behavior can be attributed to the chelation of the polysaccharide matrix by the metal ions, which hinders the polymer chain mobility as the structure becomes more compact and does not allow the water droplet to penetrate into the polymer network [40].

Figure 5A shows the changes in the water droplet outline on the different Ga (III)-CS samples with respect to time. The droplets behaved relatively similar immediately after deposition on the sample, namely the liquid beaded on the surface due to the hydrophobic nature of the polymer. With prolonged time, however, each of the water drop profiles began to change, indicating that the liquid spread out. This droplet modification might also be caused by the deformation of the film surface due to the capability of CS to retain water and swell when interacting with aqueous media. However, it is reported in the literature that the swelling behavior of CS reduces after complexation [40].

It is well documented that proteins tend to bind to hydrophobic surfaces in a higher amount and more tightly than to hydrophilic surfaces [46]. Such higher protein affinity to hydrophobic biomaterials can affect subsequent cellular responses. Thus, the prepared CS-based films are expected to be a suitable platform for cell–surface interactions.

### 3.5. Antibacterial Activity

**Figure 6:**
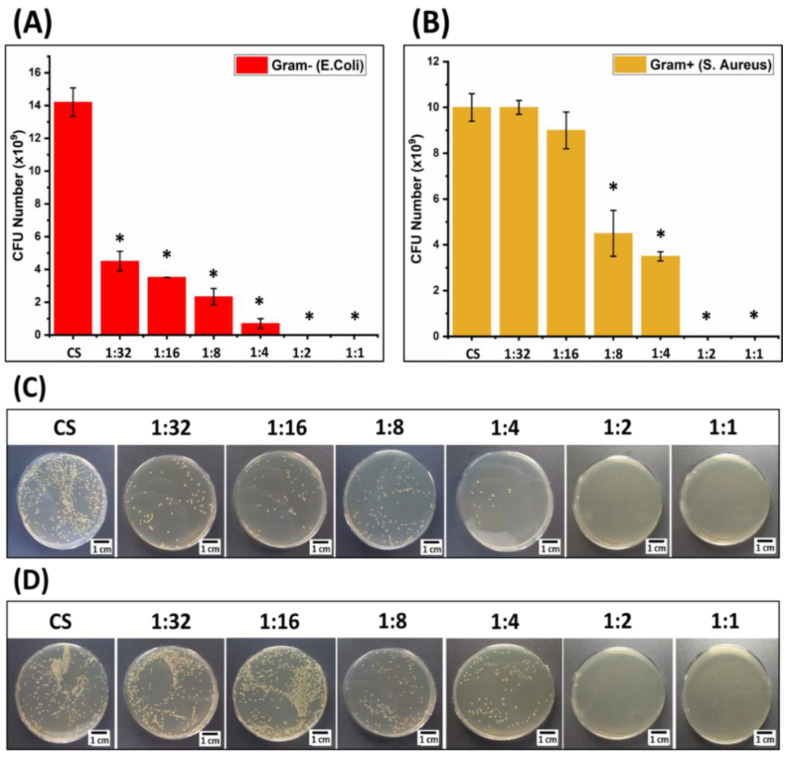
Colony forming unit (CFU) measurement of CS and Ga (III)-CS complex films after 24 h of incubation in LB medium in contact with (**A**) *E. coli* and (**B**) *S. aureus* (statistical analysis was performed with respect to pure CS samples). Photograph of the CFU of (**C**) *E. coli* and (**D***) S. aureus* after 24 h of incubation in the presence of CS and Ga (III)-CS complexes. Significant differences are represented by an asterisk (*).

The antibacterial activity of Ga (III)-CS complexes and CS against *E. coli* and *S. aureus* is presented in Figure 6. It was observed that all compositions of Ga (III)-CS complexes significantly reduced the CFU of Gram-negative (*E. coli)* compared to pure CS samples after 24 h of incubation. More specifically, 1:2 and 1:1 Ga (III)-CS complex films impaired all *E. coli* bacteria (Figure 6C). All complexes also showed antibacterial efficacy against Gram-positive (*S. aureus*) in a Ga (III) dose-dependent manner, as the samples with the two highest Ga (III) concentrations reduced the CFU number to zero (Figure 6D). As previously discussed, the antibacterial potential of the complex comes from the increased CS positive charge after chelation with metal ions and from the deleterious effect of gallium ion on bacteria [14,19,22]. Wang et al. [14] found similar results by chelating different transition metals with various charge densities. A high concentration of transition metals leads to enhanced adsorption of polycations onto the negatively charged cell surface. It is interesting to note that Ga (III)-CS (with Ga concentration below 1:2) showed slightly lower antibacterial capability against *S. aureus* than against *E. coli.* In agreement with our observation, Xu et al. [21] claimed that *S. aureus* has a much stronger anti-gallium defense system compared to *E. coli*. The discrepancy in the antibacterial effect of Ga against both bacterial species was also noticed by Valappil et al. [47], who reported that Ga(III)-doped phosphate-based glasses (PBGs) created a larger inhibition zone for *E. coli* than *S. aureus.* Moreover, the antibacterial effect of the Ga (III) protoporphyrin IX complex (PPIX) was stronger for *E. coli* (minimum inhibitory concentration (MIC) < 0.5 μg/mL) than for *S. aureus* (MIC = 1.0–2.5 μg/mL) [48]. One explanation could be the fact that Gram-positive *S. aureus* possesses a thicker cell wall than Gram-negative *E. coli*, which could possibly serve as a shielding barrier [21,49,50]. In addition, Gram-negative bacteria possess a higher negative charge that may cause an intensified attraction to metal cations, thus leading to the alteration of the cell membrane structure and permeability [51]. However, our findings suggest that the presence of sufficiently high Ga (III) concentrations in the CS-based complex provides outstanding bactericidal effects against both *E. coli* and *S. aureus* bacterial strains. In fact, an increased Ga concentration has been associated with enhanced production of reactive oxygen species (ROS) and increased expression of ROS-detoxifying enzymes, which can induce bacterial death [48,52]. Conclusively, proper antibacterial activities of Ga (III)-CS complexes could be tailored depending on the amount of metal ions incorporated during complex preparation.

### 3.6. Cell Culture Studies

**Figure 7:**
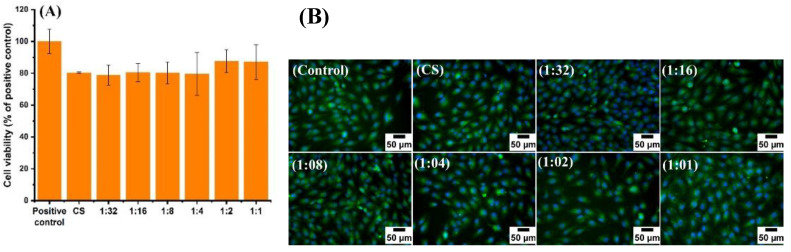
(**A**) Cell viability and (**B**) fluorescence microscope images of MG-63 cells cultured with the extracts of CS and Ga (III)-CS complexes for 24 h. The tissue culture plate was used as a control.

**Figure 8:**
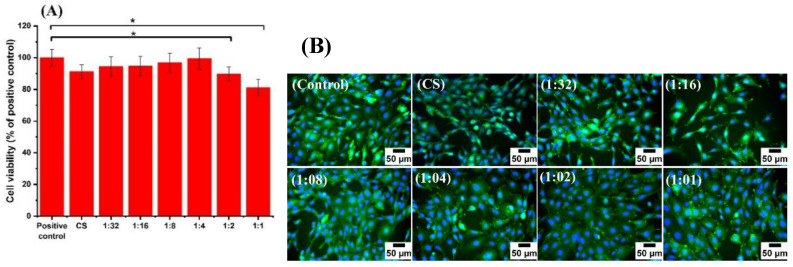
(**A**) Cell viability and (**B**) fluorescence microscope images of mouse embryonic fibroblasts (MEFs) cells cultured with the extracts of CS and Ga (III)-CS complexes for 24 h. The tissue culture plate was used as a control. Significant differences are represented by an asterisk (*).

The biocompatibility of CS has been proven in different domains of biomaterials science and biomedical engineering [53]. However, gallium can potentially exert cytotoxic effects depending upon its concentration [54]. As in the case of many pharmacological agents, gallium-based compounds should be present in sufficient concentrations at the site of biological action to generate therapeutic effects without inducing cytotoxicity. In order to evaluate the biological impact of gallium in CS-based complexes, the cell cytotoxicity of all Ga (III)-CS complexes and CS was assessed using the human osteosarcoma cell line (MG-63 cells) and mouse embryonic fibroblasts (MEFs). The results showed no statistically significant reduction of cell viability from CS and its complexes with different concentrations of Ga (III) relative to the positive control. All samples showed high relative cell viability, i.e., in the range of 80–87%, as shown in Figure 7A. The comparable cell viability values of CS and Ga (III)-CS complexes suggest that all concentrations of Ga (III) in CS did not cause a cytotoxic effect against MG-63 cells. The calculated cell viability corresponds to the fluorescence microscopy observations (Figure 7B). Moreover, cell fluorescence images of the positive control are similar to the image of the investigated samples. The cells are well spread on the surface of the well plate even in the high concentration of Ga (III) ions, i.e., the 1:1 Ga (III)-CS complex. The same behavior was observed in a previous study of Ga(III)-CS complex coatings, in which cells were placed in direct contact with the complexes [27]. From these observations, we can conclude that the studied concentrations of Ga (III) ions in Ga (III)-CS complexes do not lead to any adverse reactions in MG-63 cells. In the case of MEF cells, it can be seen that the 1:32, 1:16, 1: 8, and 1: 4 Ga (III)-CS samples did not reveal a decrease in cell viability. The higher concentration of Ga (III), i.e., in 1:2 and 1:1 Ga (III)-CS complexes, induces a reduction in cell viability, which, however, remains higher than 80% (Figure 8A). From the fluorescence microscopy images (Figure 8B), it can be observed that the cells are well distributed in the well plates, and they are not in a stressed condition even in the case of 1:2 and 1:1 Ga (III)-CS complexes. The biological response towards different metal ions is distinct. In our previous study of Cu (II)-CS complexes, it was observed that a maximum cytocompatible concentration of Cu (II) ion was between 6 and 12 mol% of free amino groups of CS. However, lower concentrations of Cu (II)-CS complexes did not show any cytotoxic effect [12]. In comparison, this study proves that the cytocompatibility of Ga (III)-CS complexes was significantly higher than the Cu (II)-CS complexes. Recently, Best et al. [52] reported the use of different polysaccharides as delivery carriers for gallium ions. The authors observed that gallium possesses a concentration-dependent cytotoxic effect, since the higher tested concentration (50 mg/mL) adversely affected human dermal neonatal fibroblast (HNDF) cells [55]. However, as confirmed by the ion release measurements, the amount of Ga (III) that is discharged from all investigated CS-based complexes in the current study is significantly lower, which explains the nontoxic behavior of Ga (III)-CS against both cell types (MG-63 and MEF).

## 4. Conclusions

Ga (III)-CS complexes with various Ga (III) contents were prepared. The influence of gallium (III) metal ions on the CS structural features, chemical composition, wettability, and biological properties was evaluated. The FTIR, XRD, and EDX results confirmed the homogeneous distribution of metal ion in the CS matrix, which supported the theory that gallium ions form coordinated complex with CS via the functional amino and hydroxyl groups. The hydrophobic nature of Ga (III)-CS complex films could be favorable for long-term cell–biomaterial interactions. The prepared films exhibited a sustained release of the therapeutic Ga ions within 14 days of immersion in PBS solution. The counting bacterial assay after 24 h of incubation showed that the antibacterial efficiency of Ga (III)-CS complex against both Gram-negative, *E. coli* and Gram-positive, *S. aureus* increased with the higher metal ion concentration. It was found that complexes exhibited excellent cell viability of mouse embryonic fibroblasts and osteoblast-like cells after 24 h of culture, which is comparable to that of pure chitosan films. We conclude that the versatile production procedure of Ga (III)-CS complexes, as well as the achieved cytocompatibility and enhanced antibacterial capability offers interesting possibilities to treat biofilm-related infections. An animal model could be considered in future investigations to obtain more information regarding the *in vivo* clinical efficacy of therapeutic modes against infections and the biological response to Ga compounds. Future research should focus on a more detailed characterization of the developed Ga (III)-CS complexes at the nanoscale and on exploring their potential biomedical applications by combining Ga (III)-CS complexes with different biomaterials (polymers, ceramics, metals) to exploit their antibacterial activity in medical devices.

## Figures and Tables

**Table 2:** Intensity ratios of Ga_Lα_ and C_k__α_ peaks of the EDX spectra of all investigated Ga (III)-CS complexes.

Ga (III): NH_2_	1:32	1:16	1:8	1:4	1:2	1:1
Ga**_Lα_**/C_k__α_ ratio [%]	3.1 ± 0.2	7.1 ± 0.3	10.5 ± 0.4	32 ± 1	37 ± 2	93 ± 2

## Supplementary Materials

The following are available online at https://www.mdpi.com/article/10.3390/pharmaceutics13101702/s1, Figure S1: Proposed chemical structure of Ga(III)-CS complexes.
