# Supplementary material for: Facile Synthesis of Gallium (III)-Chitosan Complexes as Antibacterial Biomaterial
Source: Pharmaceutics. 2021 Oct 15;13(10):1702. doi: 10.3390/pharmaceutics13101702 (PMC8541496; doi:10.3390/pharmaceutics13101702)
Supplement: Supplementary file 1 [file pharmaceutics-13-01702-s001.zip › pharmaceutics-1377567-supplementary.pdf]

pharmaceutics

Supplementary Materials: Facile Synthesis of Gallium (III)–Chitosan Complexes as Antibacterial Biomaterial

Muhammad Asim Akhtar, Zoya Hadzhieva, Kanwal Ilyas, Muhammad Saad Ali, Wolfgang Peukert and Aldo R. Boccaccini

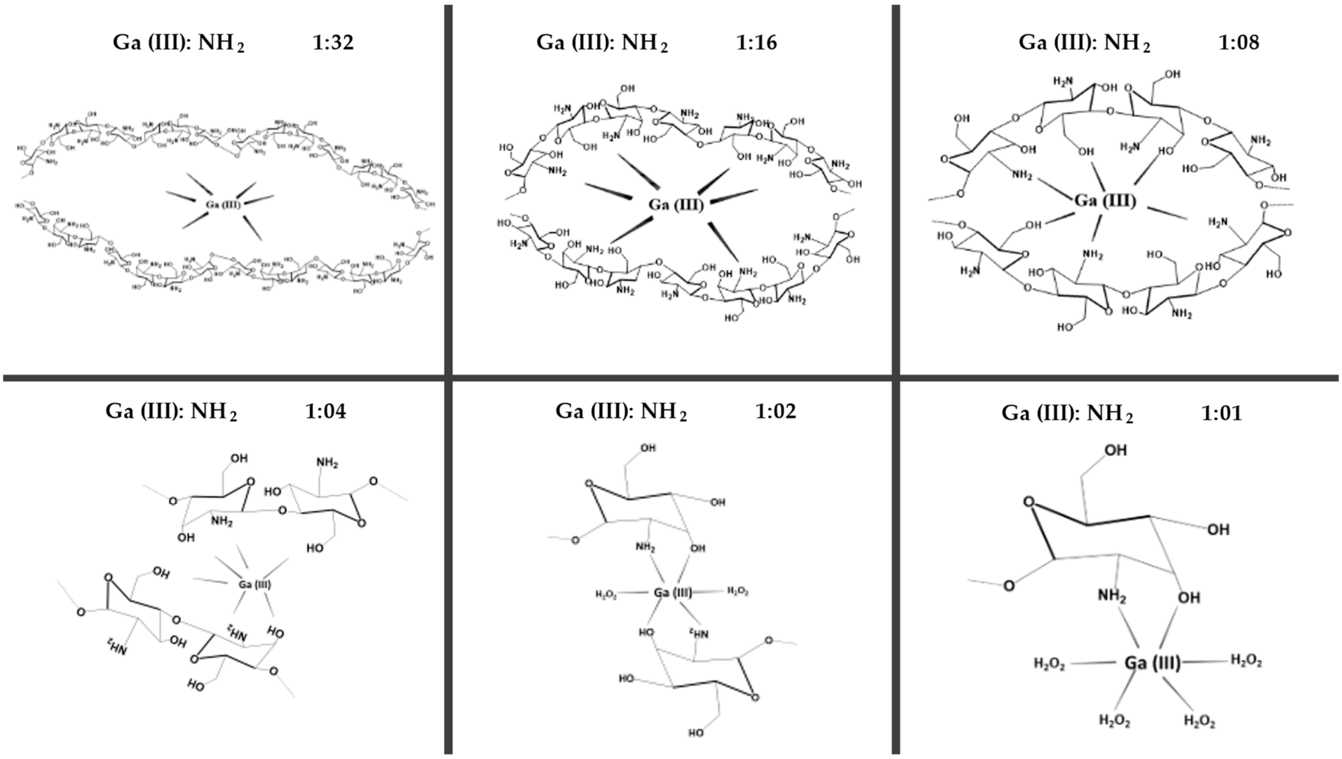

Figure S1. Proposed chemical structure of Ga(III)-CS complexes.
